# Rates of Carpal Tunnel Syndrome in a State Workers’ Compensation Information System, by Industry and Occupation — California, 2007–2014

**DOI:** 10.15585/mmwr.mm6739a4

**Published:** 2018-10-05

**Authors:** Rebecca Jackson, John Beckman, Matt Frederick, Kristin Musolin, Robert Harrison

**Affiliations:** ^1^Division of Environmental and Occupational Disease Control, California Department of Public Health, Richmond, California; ^2^Public Health Institute, Oakland, California; ^3^Hazard Evaluations and Technical Assistance Branch, National Institute for Occupational Safety and Health, CDC, Cincinnati, Ohio.

Carpal tunnel syndrome (CTS) occurs when the median nerve becomes compressed as it passes through the wrist within the carpal tunnel, resulting in pain, tingling, weakness, or numbness in the hand or the wrist. Occupational risk factors for CTS include engaging in work activities that require forceful, repetitive tasks, prolonged use of the hands or wrists in an awkward posture, or vibration (*1*). To assess trends and identify high-risk industries and occupations for CTS, the California Department of Public Health (CDPH) analyzed California workers’ compensation claims for CTS by industry (2007–2014) and occupation (2014) and calculated rates per full-time equivalent (FTE) worker. During 2007–2014, a total of 139,336 CTS cases were reported (incidence = 6.3 cases per 10,000 FTE) in California workers; the rate among women (8.2) was 3.3 times higher than that among men (2.5). Industries with the highest rates of CTS were textile, fabric finishing, and coating mills (44.9), apparel accessories and other apparel manufacturing (43.1), and animal slaughtering and processing (39.8). Industries with high rates of CTS should consider implementing intervention measures, including ergonomic evaluations and development of tools and instruments that require less repetition and force and that correct awkward postures.

In California, workers’ compensation insurance companies are required to electronically report to the Department of Industrial Relations all workers’ compensation claims for occupational injuries or illnesses that cause lost time beyond the day of injury or medical care beyond first aid. During 2007–2014, an average of 637,672 workers’ compensation claims were submitted annually (*2*). CDPH previously identified probable and possible CTS cases among these claims during 2007–2008 but undertook no further analysis of demographics or risk factors (*3*). For this analysis, all CTS cases with a date of injury during 2007–2014 were assigned a 2010 Census Industry Code by trained industry coders. CTS cases in 2014 were assigned a 2010 Census Occupation Code as a pilot of the National Institute for Occupational Safety and Health (NIOSH) Industry and Occupation Computerized Coding System computer-assisted auto-coder.* Industry- and occupation-specific rates were calculated using data from the California sample of the American Community Survey for FTE workers overall and by age group and sex.^†^ An FTE is equal to the total number of hours worked divided by 2,000 hours, which is equivalent to 50 work weeks at 40 hours per week. This accounts for different patterns of part-time work and overtime in different industries or occupations and is a measure of the risk for injury per hours worked. Changes over time were measured using rate ratios comparing industry rates during 2007–2010 and 2011–2014.

The CDPH identified 139,336 probable and possible cases of CTS; the overall rate of CTS among workers was 6.3 cases per 10,000 FTE (Table 1). The rate decreased during the study period from 6.7 during 2007–2010 to 5.9 during 2011–2014. The rate of CTS was highest among persons aged 45–54 years (8.4); the rate among women (8.2) was 3.3 times higher than that among men (2.5).

**TABLE 1:** Characteristics of carpal tunnel syndrome cases reported, by workers’ compensation claims — California, 2007–2014

Characteristic	No. of cases (%)	Rate*	Rate ratio (95% CI)
**Total**	**139,336 (100)**	**6.3**	**—**
**Age group (yrs)**			
15–24	7,143 (5)	3.1	Referent
25–34	28,583 (6)	5.0	1.6 (0.6–2.6)
35–44	35,658 (26)	6.6	2.1 (1.1–3.1)
45–54	42,682 (31)	8.4	2.7 (1.7–3.7)
55–64	22,753 (16)	7.6	2.5 (1.5–3.5)
≥65	2,240 (2)	3.6	1.2 (0.2–2.2)
**Sex**			
Male	38,403 (27)	2.5	Referent
Female	99,727 (72)	8.2	3.3 (2.3–4.3)
**Period**			
2007–2010	73,986	6.7	Referent
2011–2014	65,350	5.9	0.88 (0.85–0.91)

**Abbreviations:** CI = confidence interval; FTE = full-time equivalent.

* Carpal tunnel syndrome cases per 10,000 FTE.

Among the 20 industries with the highest rates of CTS (Table 2), three industries had CTS rates approximately six times the average rate: textile, fabric finishing, and coating mills (44.9); apparel accessories and other apparel manufacturing (43.1); and animal slaughtering and processing (39.8). Among industries with high rates of CTS, the largest numbers of CTS claims were in public administration (8,713 cases), insurance carriers (4,836), grocery stores (4,630), wired and wireless communication (3,412), and employment services (2,763). Seven industries had higher rates during 2011–2014 compared with 2007–2010; the industries with the highest relative risks during 2011–2014 compared with 2007–1010 were commercial and service industry machinery manufacturing (3.6) and knitting fabric mills (2.4).

**TABLE 2:** Number and rate of carpal tunnel syndrome (CTS) cases and relative risk, by Census Industry Code for 20 industries with the highest rates of CTS — California, 2007–2014

Industry	No. of cases	Rate*			Relative risk^†^ (95% CI)
		All years	2007–2010	2011–2014	
Textile and fabric finishing and coating mills	66	44.9	40.9	51.7	1.3 (1.1–1.4)
Apparel accessories and other apparel manufacturing	37	43.1	40.5	47.7	1.2 (1.0–1.3)
Animal slaughtering and processing	636	39.8	48.3	32.5	0.7 (0.6–0.8)
Public administration	8,713	37.5	40.3	34.8	0.9 (0.7–1.0)
Sugar and confectionery products	225	36.2	40.8	32.2	0.8 (0.7–0.9)
Employment services	2,763	36.0	31.6	39.8	1.3 (1.1–1.5)
Navigational, measuring, electro-medical, and control instruments manufacturing	979	35.1	42.4	28.4	0.7 (0.6–0.8)
Wired and wireless telecommunications	3,412	32.9	37.3	28.0	0.8 (0.6–0.9)
Aluminum production and processing	103	29.2	30.5	27.9	0.9 (0.8–1.1)
Knitting fabric mills	32	28.7	18.8	44.8	2.4 (2.0–2.8)
Insurance carriers and related activities	4,836	26.9	30.5	23.2	0.8 (0.6–0.9)
Pottery, ceramics, and plumbing fixture manufacturing	40	26.6	33.2	16.6	0.5 (0.4–0.6)
Power companies	1,701	24.3	25.4	23.3	0.9 (0.8–1.1)
Pharmaceutical and medicine manufacturing	1,229	24.2	27.7	20.9	0.8 (0.6–0.9)
Bakeries, except retail	502	22.7	24.8	21.0	0.8 (0.7–1.0)
Foundries	98	22.5	20.6	24.4	1.2 (1.0–1.4)
Software publishers	415	22.4	25.5	19.9	0.8 (0.6–0.9)
Bus service and urban transit	977	22.3	28.5	15.7	0.6 (0.5–0.7)
Dry cleaning and laundry services	572	22.1	18.9	26.0	1.4 (1.1–1.7)
Commercial and service industry machinery manufacturing	155	21.7	12.2	43.7	3.6 (2.9–4.3)

**Abbreviations**: CI = confidence interval; FTE = full time equivalent.

* CTS cases per 10,000 FTE.

^†^ The relative risk is calculated as the risk during 2011−2014 relative to the risk during 2007−2010.

The occupation categories with the highest CTS rates were production (14.0), material moving (13.4), and office and administrative support (13.0) (Table 3). The Census Occupation Codes with the highest rate of CTS were telephone operators (90.3); cafeteria, food concession, and coffee shop counter attendants (66.0); and electrical, electronics, and electromechanical assemblers (46.2).

**TABLE 3:** Rates of carpal tunnel syndrome (CTS), by occupation category and the Census Occupation Code within that category with the highest CTS rate — California, 2007–2014

Occupation category	No. of cases	Rate*	Census Occupation Code	No. of cases	Rate*
Production^†^	1,235	14.0	Electrical, electronics, and electromechanical assemblers^§^	97	46.2
Material moving^†^	558	13.4	Refuse and recyclable material collectors	22	32.2
Office and administrative support^†^	2,372	13.0	Telephone operators^§^	14	90.3
Healthcare support	315	11.9	Massage therapists	25	22.6
Building and grounds cleaning and maintenance	576	11.9	Maids and housekeeping cleaners	194	17.6
Community and social services	242	10.0	Probation officers and correctional treatment specialists	37	44.4
Protective service	316	9.6	First-line supervisors of police and detectives	28	26.2
Food preparation and serving	723	9.5	Cafeteria, food concession, and coffee shop counter attendants^§^	67	66.0
Healthcare practitioners	673	9.1	Dental hygienists	20	18.7
Legal	120	7.1	Miscellaneous legal support workers	25	13.9
Business and financial	502	6.6	Tax examiners and collectors, and revenue agents	14	35.1
Sales and related	853	6.0	Travel agents	10	15.7
Transportation and material moving	286	5.7	Bus drivers	60	14.8
Life, physical, and social science	86	5.4	Environmental scientists and geoscientists	8	13.5
Farming, fishing, and forestry	147	4.9	Graders and sorters, agricultural products	48	22.6
Installation, maintenance, and repair	198	4.9	Radio and telecommunications equipment installers and repairers	36	23.4
Computer, engineering, and science	213	4.0	Operations research analysts	48	28.3
Architecture and engineering	132	3.9	Engineering technicians, except drafters	47	9.9
Arts, design, entertainment, sports, and media	112	3.6	Miscellaneous media and communication workers	11	12.0
Construction and extraction occupations	235	3.6	Highway maintenance workers	8	19.0
Personal care and service occupations	162	3.6	Baggage porters, bellhops, and concierges	14	18.3
Management occupations	571	3.5	Medical and health services managers	53	7.7
Education, training, and library occupations	181	2.4	Library technicians	9	21.9

**Abbreviation:** FTE = full-time equivalent.

* CTS cases per 10,000 FTE.

^†^ Occupation categories with the three highest rates of CTS.

^§^ Census Occupation Codes with the three highest rates of CTS.

## Discussion

In this examination of statewide trends in demographic and occupational risk factors for CTS during 2007–2014 using California’s workers’ compensation claims, the overall incidence of CTS was 6.3 per 10,000 FTE, and the rate was approximately three times higher in women than in men. The rate decreased over time; however, this trend also mirrors a decrease in all-cause workers’ compensation claims and could be related to delayed diagnosis and reporting of CTS to workers’ compensation insurers or insurers’ misclassification of workers’ compensation CTS claims. Improved workplace ergonomic designs and employment demographic shifts might have contributed to this trend. Industries with high rates of CTS included those that manufacture apparel, process food, and perform administrative work. The occupation groups with the highest rates included production workers, material moving workers, and office and administrative support workers. Workers in these occupations are often required to perform forceful or repetitive tasks with their hands (e.g., sewing clothing, butchering meat, or repeatedly lifting heavy items), or maintain an awkward posture on the job (e.g., driving a motor vehicle, working on a production line, or computer work), all known risk factors for CTS.

These findings are consistent with those previously reported using data from the 2010 National Health Interview Survey, which estimated that the prevalence of CTS was higher among women, and that the highest ratios of CTS cases to percentage of workforce were among production, office and administrative support, and personal care and service occupations (2.5%, 1.66%, and 1.53%, respectively) (*4*). A study using Washington State workers’ compensation claims reported an annual decrease of 6.2% CTS incidence during 2002–2013, similar to the decrease in this analysis (*5*). A pooled analysis of six prospective cohorts documented CTS incidence among 50 workgroups of 2.3 cases per 100 person-years, which is higher than the incidence estimates in this analysis (*6*). This could indicate that CTS is underdiagnosed or underreported by workers or employers, or that health care providers outside of the workers’ compensation system are treating cases of work-related CTS. Costs for CTS medical care are estimated to be $2 billion annually in the United States, primarily from surgical releases; nonmedical costs (e.g., for mental or psychological health treatment, loss of earnings and productivity, and costs for legal services) are estimated to be much higher (*7*).

These results suggest that workers’ compensation claims data can be a useful tool to identify industries and occupations where workers are at risk for developing CTS. Workers’ compensation data can help describe work-related injuries like CTS that might be underreported in other systems and provide case-level demographic and risk factor data that might not be available from other estimates (*8*). Workplace ergonomic interventions that modify tasks, workstations, tools, and equipment can decrease known ergonomic hazards and prevent workplace injuries, including CTS. However, it is not known whether ergonomic interventions were implemented or maintained within the industries with high rates of CTS during the study period.

The findings in this report are subject to at least four limitations. First, inconsistent industry coding and lack of standard occupation coding create difficulties in identifying risk factors within workers’ compensation systems. As noted, the NIOSH Industry and Occupation Computerized Coding System auto-coder, first released for public use in December 2012, facilitated the occupation coding used in this analysis. As auto-coding algorithms improve, more rapid identification of industries and occupations will be possible. Second, because the California workers’ compensation claims system does not collect race and ethnicity data, it was not possible to calculate rates for these important demographic variables. Third, California does not collect the number of employees’ hours worked by industry, so the American Community Survey was used to calculate FTEs. Using FTEs is a more accurate representation of the risk in these industries than number of workers because it includes time at risk for injury similarly in industries with different percentages of part-time and overtime workers. These three limitations present challenges to analyzing a data set designed for administrative rather than public health surveillance use. Finally, only 1 year of data (2014) was occupation coded because of the time and resources necessary to code occupation, even with the assistance of the NIOSH Industry and Occupation Computerized Coding System. Whether the occupations at risk for CTS changed over time is not addressed by this analysis.

Analysis of workers’ compensation records is helpful for understanding the industries and occupations that are at a higher risk for CTS and for determining allocation of limited resources for prevention. Industries and occupations identified with high rates of CTS should consider implementing intervention measures, including ergonomic evaluations and development of tools and instruments that require less repetition and force and correct awkward postures. States could use their workers’ compensation data to identify cases of CTS and use this information to target prevention activities.

**Summary**

**What is already known about this topic?**

Carpal tunnel syndrome (CTS) is an important contributor to work-related disability.

**What is added by this report?**

Workers’ compensation claims of CTS in California during 2007–2014 overall were 6.3 per 10,000 full-time equivalent workers. Female workers and workers in industries that manufacture apparel, process food, and perform administrative work were at highest risk for CTS. The highest rates of CTS were among telephone operators; cafeteria, food concession, and coffee shop counter attendants; and electrical, electronics, and electromechanical assemblers.

**What are the implications for public health practice?**

Industries with high rates of CTS should consider implementing intervention measures, including ergonomic evaluations and development of tools and instruments that require less repetition and force and correct awkward postures.

## Footnotes

*
https://wwwn.cdc.gov/niosh-nioccs/ .

† https://www.census.gov/programs-surveys/acs/.
